# Supplementary material for: Monte Carlo study of the pseudogap and superconductivity emerging from quantum magnetic fluctuations
Source: Nat Commun. 2022 May 12;13:2655. doi: 10.1038/s41467-022-30302-x (PMC9098861; doi:10.1038/s41467-022-30302-x)
Supplement: Supplementary file 1 — Supplementary Information [file 41467_2022_30302_MOESM1_ESM.pdf]

Supplementary Information for : Monte Carlo study of the pseudogap and superconductivity emerging from quantum magnetic fluctuations

In this supplementary information, we present the details of quantum Monte Carlo (QMC) implementation and more results of the phase diagram in different phases, as well as the theoretical analysis upon the QMC data.

Supplementary Note 1: Details of QMC simulation.

A. Quantum Rotor Model.

The Monte Carlo simulation on the quantum rotor model (QRM) starts from employing a proper basis for the Hamiltonian. As shown in Eq. 2 in main text, the boson part has global U(1) symmetry under θ representation. We adopt the representation of the angle variable θ for each site, ranging between $[0, 2\pi)$, which are the eigenstates of the potential part. Using the canonical commutation relation $[\hat{\theta}_i, \hat{n}_j] = i\delta_{i,j}$, the QRM Hamiltonian can be expressed as,

$$H_{\text{qr}} = \hat{T} + \hat{U} \tag{1}$$

$$= \frac{U}{2}\sum_i \big(-i\frac{\partial}{\partial\hat{\theta}_i}\big)^2 - t_{\text{b}}\sum_{\langle i,j\rangle}\cos(\hat{\theta}_i - \hat{\theta}_j) \tag{2}$$

and the partition function obeys,

$$Z = \text{Tr}\left\{ e^{-\beta[-\frac{U}{2}\sum_i \frac{\partial^2}{\partial\hat{\theta}_i^2} - t_{\text{b}}\sum_{\langle i,j\rangle}\cos(\hat{\theta}_i - \hat{\theta}_j)]} \right\} \tag{3}$$

Using Trotter decomposition, we can divided β into M slices with step $\Delta\tau = \beta/M$, and insert the complete sets of the $\{\theta_i\}$ at each time slice. We have,

$$Z = \int \mathcal{D}\theta \prod_{l=0}^{M-1} \langle\{\theta(l+1)\}|e^{-\Delta\tau\hat{T}}e^{-\Delta\tau\hat{V}}|\{\theta(l)\}\rangle \tag{4}$$

The states follow the periodic boundary condition $\{\theta(M)\} = \{\theta(0)\}$. For the potential part, $\theta_i(l)$-s are the eigenstates of V and can be directly calculated. For the kinetic part, if one inserts a complete basis of $J_i(l)$ as the integer-valued angular momentum at site i and time slice l, one finds that

$$T(l) = \sum_{\{J\}}\prod_i e^{-\frac{\Delta\tau U}{2}[J_i(l)]^2}\langle\theta_i(l+1)|J_i(l)\rangle\langle J_i(l)|\theta_i(l)\rangle, \tag{5}$$

The term $\langle\theta_i(l)|J_i(l)\rangle$ has a complex value $e^{iJ_i(l)\theta_i(l)}$. Next, we transfer the square term of $J_i(l)$ into linear term with the help of the Poisson summation formula,

$$\begin{aligned} T(l) &= \prod_i\sum_J e^{-\frac{\Delta\tau U}{2}J^2}e^{iJ(\theta_i(l)-\theta_i(l+1))} \\ &= \prod_i\sum_{m=-\infty}^{\infty}\int_{-\infty}^{\infty} dJ e^{2\pi iJm}e^{-\frac{\Delta\tau U}{2}J^2}e^{iJ(\theta_i(l)-\theta_i(l+1))} \\ &= \prod_i\sum_{m=-\infty}^{\infty}\sqrt{\frac{2\pi}{\Delta\tau U}}e^{-\frac{1}{2\Delta\tau U}(\theta_i(l)-\theta_i(l+1)-2\pi m)^2}. \end{aligned} \tag{6}$$

Then, we modify this by a Villain approximation to

$$T(l) \approx \prod_i e^{\frac{1}{\Delta\tau U}\cos(\theta_i(l)-\theta_i(l+1))} \tag{7}$$

where the kinetic part of QRM can be regarded as the effective interaction along imaginary time axis. We finally map the QRM to 3D anisotropic XY model [1], and the space-time configuration of the rotors, as shown in the Fig. 1a of the main text, plays the role of the usual auxiliary field for the determinant QMC simulations, which we will discuss next.

B. Determinantal QMC implementations.

The determinantal quantum Monte Carlo (DQMC) is designed to deal with the interacting fermion lattice model with quartic interactions [2] and to decouple the quartic interactions into auxiliary bosonic fields coupled with fermion bilinears. In recent years, there new developments allow one to bestow the bosonic auxiliary field with a bosonic Hamiltonian and investigate the situation of the critical bosons coupled with various Fermi surface geometries [3], which is the path we take in this work.

In DQMC, one transfers the non-eigenstates to a series of classical configurations, such as the space-time rotor configurations in previous section, and then samples in the resulting configuration space. To start with, one writes down the path integral of partition function,

$$Z = \mathrm{Tr}\{e^{-\beta \hat{H}}\} = \mathrm{Tr}\{\prod_{m=1}^{M} e^{-\Delta\tau \hat{H}}\} \tag{8}$$

Here, $M = \beta/d\tau$, denoting the number of the imaginary time slices. $\hat{H}$ is the total Hamiltonian and contains both bosonic and fermionic parts, and their interaction. The trace operation can be divided into trace for fermions Tr_{F} and bosons $\int d\theta_i$, where we express the bosonic degrees of freedom as θ for each site. Next, we insert a series of unit operators with periodic boundary conditions $\{\theta(M)\} = \{\theta(0)\}$ and make $\Delta\tau \to 0$,

$$Z = \mathrm{Tr}_{\mathrm{F}}\left\{\int \mathcal{D}\theta \langle\{\theta\}|\prod_{l=0}^{M-1} e^{-\Delta\tau\hat{H}}|\{\theta\}\rangle\right\} \tag{9}$$

$$= \mathrm{Tr}_{\mathrm{F}}\left\{\int \mathcal{D}\theta \prod_{l=0}^{M-1} \langle\{\theta(l+1)\}|e^{-\Delta\tau\hat{H}_{\mathrm{qr}}}e^{-\Delta\tau\hat{H}_{\mathrm{F}}}e^{-\Delta\tau\hat{H}_{\mathrm{qr-f}}}|\{\theta(l)\}\rangle\right\} \tag{10}$$

Next, utilizing the Supplementary Equation (7), we write the partition function as

$$Z = \int \mathcal{D}\theta \left(\prod_{l=0}^{M-1}\prod_{i} e^{\frac{1}{\Delta\tau U}\cos(\theta_i(l)-\theta_i(l+1))}\right)\left(\prod_{l=0}^{M-1} e^{\Delta\tau t_b \sum_{\langle i,j\rangle}\cos(\theta_i(l)-\theta_j(l))}\right)\mathrm{Tr}_{\mathrm{F}}\left\{\prod_{l=0}^{M-1} e^{-\Delta\tau\hat{H}_{\mathrm{F}}}e^{-\Delta\tau\hat{H}_{\mathrm{qr-f}}}\right\} \tag{11}$$

The bosonic part can be taken out of the fermion trace. Furthermore, the kinetic part of free fermion is independent of configuration and can be calculated at the beginning of the simulation, while the interaction part of boson and fermion depends on the configurations of θ. The calculation process on Tr_{F} is always displayed as the determinant. Finally, the total weight of configuration is,

$$\begin{aligned} Z &= \int \mathcal{D}\theta\, \mathrm{W_b}(\{\theta\}) \det(\mathbf{1} + \prod_{l=0}^{M-1} e^{-\Delta\tau H_{\mathrm{F}}}e^{-\Delta\tau H_{\mathrm{qr-f}\{\theta(l)\}}}) \\ &= \int \mathcal{D}\theta\, \mathrm{W_b}(\{\theta\}) \det(\mathbf{1} + B(\beta,0)_{\{\theta\}}) \\ &= \int \mathcal{D}\theta\, \mathrm{W_b}(\{\theta\})\mathrm{W_F}(\{\theta\}) \end{aligned} \tag{12}$$

where $\mathrm{W_b}(\{\theta\})$ is the weight of bosonic part, and $H_{\mathrm{F}}, H_{\mathrm{qr-f}}$ is the matrix in fermionic layer, spin, coordinate representation. So far, we have mapped the model to a series of classical configurations and obtained its weight. Using Markov chain, we sample the configuration of $\{\theta\}$ and implement both local update schemes and global updates - a Wolff update scheme - to avoid critical slowing down, see algorithm analysis and details of QRM in [1].

C. QMC sign problem.

We find an antiunitray transformation $\mathcal{K} = i\sigma_y K$ under which the model is invariant, where σ_y is a Pauli matrix in the layer basis, and K is the complex conjugation operator. Thus the model is free of the sign problem, and

$$\mathrm{W_F} = \det(\mathbf{1} + B'(\beta, 0)_{\{\theta\}})^2 \tag{13}$$

B' is $2N \times 2N$ dimension matrix for single layer fermions, where $N = L \times L$ is the number of sites. Since the model is symmetric for two layers, the Green's function is the same for both layers with same site and spin index, i.e. $G_{11}^{\sigma\sigma'} = G_{22}^{\sigma\sigma'}$, where $G_{\lambda\lambda'}^{\sigma\sigma'} = \langle T \hat{c}_{i\sigma\lambda} \hat{c}_{j\sigma\lambda'}^{\dagger} \rangle$.

D. Controlling finite-size effects.

The simulation of DQMC is restricted to finite system sizes, and its computational complexity scales as $O(\beta N^3)$ [3]. Therefore, it is necessary to reduce the finite size effect in simulations as much as possible. To increase the momentum resolution on finite size simulations, we introduce a magnetic field perpendicular to the lattice plane called z-direction flux. The magnetic field changes the dispersion relation of the free system to mimic the DOS of the infinite system [4]. Since the lattice site is finite, the flux must be quantized. The magnetic field is introduced via the Peirls phase factors on the bonds,

$$\hat{c}_{i\sigma\lambda}^{\dagger} \hat{c}_{j\sigma\lambda} \rightarrow e^{i \int_{\mathbf{r}_i}^{\mathbf{r}_j} \mathbf{A}_{\sigma\lambda}(\mathbf{r}) d\mathbf{r}} \hat{c}_{i\sigma\lambda}^{\dagger} \hat{c}_{j\sigma\lambda} = e^{iA_{ij}} \hat{c}_{i\sigma\lambda}^{\dagger} \hat{c}_{j\sigma\lambda} \tag{14}$$

with $\mathbf{B} = \nabla \times \mathbf{A}$ and Φ_0 the flux quanta. We take the Landau gauge $\mathbf{A}(\mathbf{r}) = -B(y, 0, 0)$, which is independent of spin and layer index. To satisfy the periodic boundary condition, the boundary hopping terms must have different form compared with that of the inner bonds. Furthermore, we hope the flux on each area of lattice plane is equivalent. Since the model has the next-nearest hopping term, the square area encircled by four adjacent sites can be divided into four triangular parts. We design the magnetic field to satisfy this condition and for the nearest-neighbor hopping the phases A_{ij} read,

$$A_{ij} = \begin{cases} +\dfrac{2\pi}{\phi_0} B \cdot i_y, \leftarrow \text{hopping} \\ -\dfrac{2\pi}{\phi_0} B \cdot i_y, \rightarrow \text{hopping} \\ 0, \uparrow, \downarrow \text{hopping} \\ +\dfrac{2\pi}{\phi_0} B \cdot L \cdot i_x, \uparrow \text{hopping} \\ -\dfrac{2\pi}{\phi_0} B \cdot L \cdot i_x, \downarrow \text{hopping} \end{cases} \tag{15}$$

For the next-nearest-neighbor hopping,

$$A_{ij} = \begin{cases} +\dfrac{2\pi}{\phi_0} B \cdot i_y, \swarrow \text{ hopping} \\ -\dfrac{2\pi}{\phi_0} B \cdot i_y, \nearrow \text{ hopping} \\ +\dfrac{2\pi}{\phi_0} B \cdot i_y, \nwarrow \text{ hopping} \\ -\dfrac{2\pi}{\phi_0} B \cdot i_y, \searrow \text{ hopping} \\ +\dfrac{2\pi}{\phi_0} B \cdot (L i_x - i_y), \swarrow \text{ hopping(boundary crossing)} \\ -\dfrac{2\pi}{\phi_0} B \cdot (L i_x - i_y), \nearrow \text{ hopping(boundary crossing)} \\ +\dfrac{2\pi}{\phi_0} B \cdot (L i_x + i_y), \nwarrow \text{ hopping(boundary crossing)} \\ -\dfrac{2\pi}{\phi_0} B \cdot (L i_x + i_y), \searrow \text{ hopping(boundary crossing)} \end{cases} \tag{16}$$

where $B = \frac{\Phi_0}{L^2}$ is the unit magnetic flux, and i_x, i_y are the indices of site range between 1 and L in the x and y lattice directions. Various arrows represent the direction of hopping terms from site i to j. Note that when $L \to \infty$, the magnetic field approaches 0, and the Hamiltonian goes back to the original one. The method of adding z-flux significantly reduces finite size effects. However, the magnetic field breaks the translation symmetry, i.e., the momentum k is not valid for fermion. In the DQMC simulation, we add the z-flux when measuring bosonic observables, e.g., bosonic susceptibility. While for fermionic observables e.g. spectral functions, superfluid density, we drop it.

Supplementary Table 1. **Various explored pairing channels.**

Channel	Description	Definition
$C_{\text{os,is}}$	On-site, s-wave, Intra-layer, spin-singlet	$\frac{1}{\sqrt{2}}(\hat{c}_{i1\uparrow}\hat{c}_{i1\downarrow} + \hat{c}_{i1\downarrow}\hat{c}_{i1\uparrow})$
$C_{\text{os,ts}}$	On-site, s-wave, layer-triplet, spin-singlet	$\frac{1}{\sqrt{2}}(\hat{c}_{i1\uparrow}\hat{c}_{i2\downarrow} - \hat{c}_{i1\downarrow}\hat{c}_{i2\uparrow})$
$C_{\text{os,st0}}$	On-site, s-wave, layer-singlet, spin-triplet($S=0$)	$\frac{1}{\sqrt{2}}(\hat{c}_{i1\uparrow}\hat{c}_{i2\downarrow} + \hat{c}_{i1\downarrow}\hat{c}_{i2\uparrow})$
$C_{\text{os,st1}}$	On-site, s-wave, layer-singlet, spin-triplet($S=1$)	$\frac{1}{\sqrt{2}}(\hat{c}_{i1\uparrow}\hat{c}_{i2\uparrow} - \hat{c}_{i2\uparrow}\hat{c}_{i1\uparrow})$
$C_{\text{ns,is}}$	Nearest-neighbor, s-wave, Intra-layer, spin-singlet	$\frac{1}{\sqrt{8}}\sum_l f_{ns}(\delta_l)(\hat{c}_{i1\uparrow}\hat{c}_{i+\delta_l 1\downarrow} + \hat{c}_{i1\downarrow}\hat{c}_{i+\delta_l 1\uparrow})$
$C_{\text{ns,ts}}$	Nearest-neighbor, s-wave, layer-triplet, spin-singlet	$\frac{1}{\sqrt{8}}\sum_l f_{ns}(\delta_l)(\hat{c}_{i1\uparrow}\hat{c}_{i+\delta_l 2\downarrow} - \hat{c}_{i1\downarrow}\hat{c}_{i+\delta_l 2\uparrow})$
$C_{\text{ns,st0}}$	Nearest-neighbor, s-wave, layer-singlet, spin-triplet($S=0$)	$\frac{1}{\sqrt{8}}\sum_l f_{ns}(\delta_l)(\hat{c}_{i1\uparrow}\hat{c}_{i+\delta_l 2\downarrow} + \hat{c}_{i1\downarrow}\hat{c}_{i+\delta_l 2\uparrow})$
$C_{\text{ns,st1}}$	Nearest-neighbor, s-wave, layer-singlet, spin-triplet($S=1$)	$\frac{1}{\sqrt{8}}\sum_l f_{ns}(\delta_l)(\hat{c}_{i1\uparrow}\hat{c}_{i+\delta_l 2\uparrow} - \hat{c}_{i1\uparrow}\hat{c}_{i+\delta_l 2\uparrow})$
$C_{\text{np,it0}}$	Nearest-neighbor p_x-wave, Intra-layer, spin-triplet($S=0$)	$\frac{1}{\sqrt{4}}\sum_l f_{np}(\delta_l)(\hat{c}_{i1\uparrow}\hat{c}_{i+\delta_l 1\downarrow} + \hat{c}_{i1\downarrow}\hat{c}_{i+\delta_l 1\uparrow})$
$C_{\text{np,it1}}$	Nearest-neighbor p_x-wave, Intra-layer, spin-triplet($S=1$)	$\frac{1}{\sqrt{2}}\sum_l f_{np}(\delta_l)\hat{c}_{i1\uparrow}\hat{c}_{i+\delta_l 1\uparrow}$

Supplementary Note 2: Physical observables.

In order to obtain the phase diagram of our model in the main text, we measure different physical observables in DQMC simulations, and analyze their behavior. Besides the main results presented in the main text, here we give a detailed description of the rest of them.

A. Pairing susceptibility and superfluid density.

Superconductivity is expected to be enhanced near the QCP [5–7], but the detailed competition of the pseudogap, nFL and superconductivity in our system still needs to be revealed with different physical ob-

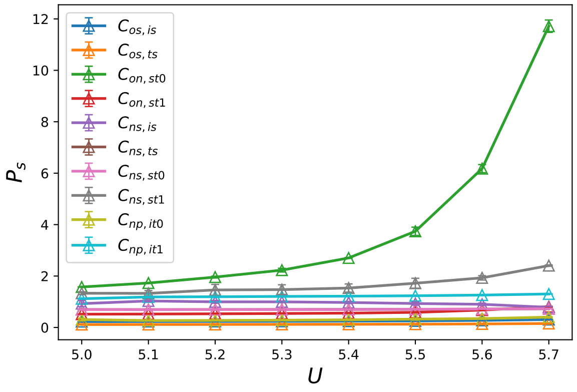

Supplementary Figure 1. Comparision of different pairing channels versus U. Pairing susceptibility of various pairing channels versus U of $\beta = 16$ of system size $L = 12$. The green line corresponds to the orbital-singlet, spin-triplet channel, which is the putative dominant channel. When U gets larger and approaches superconducting region, only $C_{\text{os,st0}}$ enhances remarkablely.

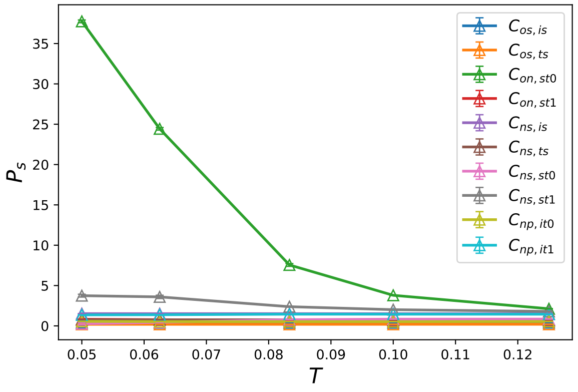

Supplementary Figure 2. Comparision of different pairing channels versus T. Pairing susceptibility various pairing channels versus T of $U = 6.0$ of system size $L = 12$. The green line corresponds to the orbital-singlet, spin-triplet channel, which is the putative dominant channel. When T gets lower and enters superconducting region, only $C_{\text{os,st0}}$ enhances remarkablely.

servables.

As for the pairing, considering that the interaction is on-site and layer and spin symmetric, we compute a number of different on-site channels and find the strongest one occurs in the s-wave channel with orbital-singlet and spin-triplet $C_{\text{os,st0}}$, with the order parameter

$$\Delta(\mathbf{r}) = C_{\text{os,st0}} = \frac{1}{\sqrt{2}}(\hat{c}_{\mathbf{r}1\uparrow}\hat{c}_{\mathbf{r}2\downarrow} + \hat{c}_{\mathbf{r}1\downarrow}\hat{c}_{\mathbf{r}2\uparrow}) \quad (17)$$

where 1,2 are layer indices. The detailed definition of the various pairing channels is listed in Supplementary Table 1. Note $\hat{x}, \hat{y}$ represent unit vector along positive x, y direction. $f_{\text{ns,is}}(\delta_l) = 1$, for $\delta_l = \hat{x}, \hat{y}$ and -1, for $\delta_l = -\hat{x}, -\hat{y}$. $f_{\text{np,is}}(\delta_l) = 1$, for $\delta_l = \hat{x}$, and -1 for $\delta_l = -\hat{x}$.

We construct the pairing susceptibility defined as,

$$P_{\text{s}} = \frac{1}{L^2}\int_0^\beta \sum_i (\Delta^\dagger(\mathbf{r}_i, \tau)\Delta(\mathbf{0}, 0)). \quad (18)$$

P_{s} captures the dynamic pair-pair correlation, which increases as temperature goes down. We show the results for all pairing channels and find no response therein in Supplementary Figure 1 and Figure 2 for other channels, except $C_{\text{os,st0}}$. Since the SC pair has quasi-long-range order below $T_{\text{c}}(T_{\text{KT}})$, P_{s} will exhibit scaling behavior with system size as $P_{\text{s}} = L^{2-\eta} f(L \cdot \exp(-\frac{A}{(T-T_{\text{c}})^{1/2}}))$ as T approches T_{c} from above, and thus at $P_{\text{s}} \propto L^{2-\eta}$ with $\eta_{\text{KT}} = 1/4$ at the transition at the thermodynamic limit [8–10]. We show the evolution pairing susceptibility of various pairing channels approaching QCP in Supplementary Figure 1 and Figure 2, from which one reads only s-wave orbital-singlet and spin-triplet channel plays the crucial role of superconductivity.

Further supporting evidence for the establishment of quasi-long-range order of s-wave pairing is the superfluid density ρ_{s}. ρ_{s} describes tendency towards pairing, and is regarded as a measure of the ratio of superconducting electron density over compared to all the itinerant electrons [11, 12]. The method to calculate ρ_{s} is derived from the linear response to an external magnetic field. Adding a vector potential $A_x(\mathbf{r}_i, t)$ with harmonic frequency ω to the bond of the free fermion system and expanding to second order, one can deduce that the total induced current density $J_x(\mathbf{q}, \omega)$ is,

$$\langle J_x(\mathbf{q}, \omega)\rangle = -\left[\langle -k_x\rangle - \Lambda_{xx}(\mathbf{q}, \omega)\right] A_x(\mathbf{q}, \omega) \quad (19)$$

where k_x is the kinetic energy density. $\Lambda_{xx}(\mathbf{q}, \omega)$ is the current-current correlation function, which is associated with the paramagnetic current density $j_x^\sigma(\mathbf{r}_i, \tau)$,

$$\Lambda_{xx}(\mathbf{q}) = \frac{1}{4}\sum_{i\sigma\sigma'}\int_0^\beta d\tau e^{-i\mathbf{q}\mathbf{r}_i}\langle j_x^\sigma(\mathbf{r}_i, \tau) j_x^{\sigma'}(0, 0)\rangle \quad (20)$$

where $j_x^\sigma(\mathbf{r}_i,\tau)$ is defined as,

$$
\begin{aligned}
&j_x^\sigma(\mathbf{r}_i,\tau)\\
&= it_1 \sum_{\lambda,\sigma} \left(\hat{c}^\dagger_{i,\lambda\sigma}(\tau)\hat{c}_{i+\hat{x},\lambda\sigma}(\tau) - \hat{c}^\dagger_{i,\lambda\sigma}(\tau)\hat{c}_{i-\hat{x},\lambda\sigma}(\tau)\right)\\
&+ it_2 \sum_{\lambda,\sigma} \left(\hat{c}^\dagger_{i,\lambda\sigma}(\tau)\hat{c}_{i+\hat{x}+\hat{y},\lambda\sigma}(\tau) - \hat{c}^\dagger_{i,\lambda\sigma}(\tau)\hat{c}_{i-\hat{x}+\hat{y},\lambda\sigma}(\tau)\right)\\
&+ it_2 \sum_{\lambda,\sigma} \left(\hat{c}^\dagger_{i,\lambda\sigma}(\tau)\hat{c}_{i+\hat{x}-\hat{y},\lambda\sigma}(\tau) - \hat{c}^\dagger_{i,\lambda\sigma}(\tau)\hat{c}_{i-\hat{x}-\hat{y},\lambda\sigma}(\tau)\right)
\end{aligned}
\tag{21}
$$

The criteria of superconductivity comes from the Meissner effect that if the current density response of a superconductor in a static, $\omega = 0$, long wavelength $q_y = 0$, the London equation is given by,

$$J_x(q_y) = -\rho_\mathrm{s} A_x(q_y) \tag{22}$$

where, ρ_s is the superfluid density to be calculated. The response is always transverse such that one can take the different order of the long wavelength transverse and longitudinal limit and obtain,

$$
\begin{aligned}
\rho_\mathrm{s} &= \langle -k_x\rangle - \Lambda_{xx}(q_x = 0, q_y \to 0, i\omega = 0)\\
0 &= \langle -k_x\rangle - \Lambda_{xx}(q_x \to 0, q_y = 0, i\omega = 0)
\end{aligned}
\tag{23}
$$

which means ρ_s can be calculated from the current-current correlation function. In the thermodynamic limit, one expects that ρ_s has a universal jump at the transition point, which according to the renormalization theory of the BKT phase transition [13] is $\rho_\mathrm{s} = \frac{2T_\rho}{\pi}$. We thus obtain T_ρ by plotting $\rho_\mathrm{s}(T)$, and looking for the crossing of ρ_s with $\frac{2T}{\pi}$. It is also noted that in the correlated electron systems, such crossing temperature actually tends to overestimate the T_c compared with that obtained from the pairing susceptibility [8, 9], we have also confirmed such behavior in our simulation.

B. Bosonic susceptibilities.

In the DQMC, we compute the dynamic bosonic susceptibility

$$\chi(h, T, \mathbf{q}, \omega_n) = \frac{1}{L^2}\int d\tau \sum_{ij} e^{i\omega_n\tau - i\mathbf{q}\mathbf{r}_{ij}} \langle\theta_i(\tau)\theta_j(0)\rangle. \tag{24}$$

For the bare rotor model, the behavior of dynamic susceptibility has the standard form,

$$\chi_0(\mathbf{q}, \omega_n) = \frac{1}{\omega_n^2 + q^2 + \xi_\mathrm{c}^{-2}}, \tag{25}$$

where ξ_c is the correlation length of bosonic field, which diverges at the critical point (in the lattice simulation such divergence is parameterized by the inverse distance towards the QCP in terms of the control parameter of the transition such as $U - U_\mathrm{c}$). When taking $q = 0, \omega_n = 0$, the dynamic susceptibility goes back to the uniform static susceptibility χ, which only depends on temperature and tuning parameter U. Our results of the uniform static susceptibility of the coupled system can not be captured by generic the Curie-Weiss form in Supplementary Equation (25). Non-monotonous behaviour versus temperature is observed with fixing U and reducing T in a wide parameter range in the phase diagram (for example, as will be discussed in Supplementary Figure **4**). This is the interesting fact that the coupling has altered the nature of the scaling in QCP and in our case such behavior serves as a signature of substantial superconducting fluctuations.

As shown in the Fig. 4 in the main text, the ω_n-dependence dynamic susceptibility follows a non-conserved bosonic order rule [14], and displays continuous behavior at $\omega_n = 0$, from which the novel quantum critical scaling behavior of our system is fully revealed. In the DQMC simulations, we also explore q-dependence and show the results in the following section.

Supplementary Note 3: Pseudogap, superconductivity and the representative scans in the phase diagram.

A. Scan at the maximum T_c.

We start with the scan at $U = 6$ as a function of reducing temperature. In Fig. 2a in the main text, $N(\omega)$ is presented at various temperatures. It is clear that there emerges a pseudogap at the temperature $T = 0.1$ ($\beta = 10$), and as the temperature is further reduced, the pseudogap steadily widens, and at the temperature of $T = 0.05$ ($\beta = 20$), the full gap opens in the single-particle spectrum.

These two temperature/energy scales of the pseudogap region, are consistent with that in other physical observables. Supplementary Figure **3** shows $\rho_\mathrm{s}(T)$ along the same scan, and one sees that the crossing temperature for larger system sizes are at the onset of the pseudogap temperature $T = 0.1$. Supplementary Figure **4** shows the uniform static susceptibility, and one observes a clear suppression of χ, i.e. the deviation from the Curie-Weiss behavior is due to the onset of superconducting fluctuations, at the same temperature scale of $T = 0.1$. As the pseudogap spectra gradually evolve into a full gap when temperature is decreasing, the superconducting fluctuation becomes stronger, and eventually renders the system into the quasi-long-

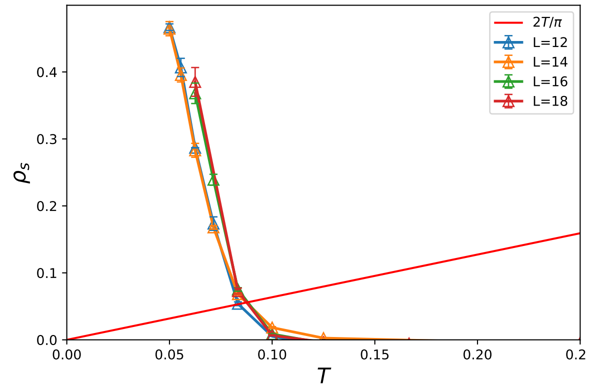

Supplementary Figure 3. **Superfluid density at the maximum $T_{\mathbf{c}}$.** Superfluid density ρ_{s} versus temperature at $U = 6$ for system sizes $L = 12, 14, 16, 18$. The onset temperature of SC fluctuation T_ρ is approximated by the crossover temperature for curve of $\rho_{\mathrm{s}}(L \to \infty)$ and linear function with slope $\frac{2}{\pi}$. For $L = 12, 14$, such temperature is at the scale of $T \sim 0.1$, consistent with the onset of pseudogap in Fig. 2a in main text.

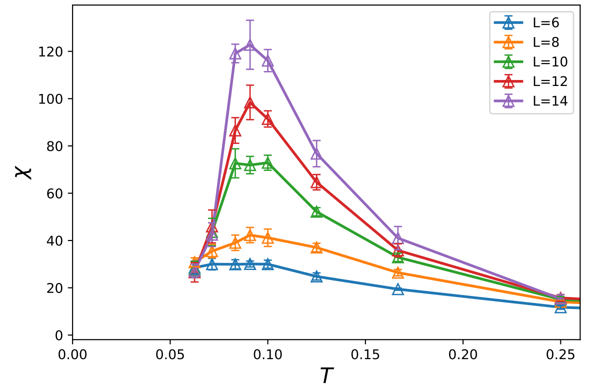

Supplementary Figure 4. **Bosonic static susceptibility at the maximum $T_{\mathbf{c}}$.** Bosonic static susceptibility $\chi(\mathbf{q} = 0, \omega = 0)$ versus temperature at $U = 6$ for system sizes $L = 6, 8, 10, 12, 14$. Considering finite size effect, the nonmonotonous behavior of $\chi(T)$ depicts a crossover of two phase, whose transition temperature is approxiametly at $T = 0.09$.

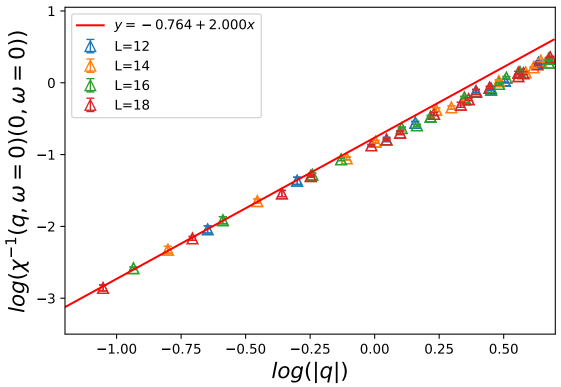

Supplementary Figure 5. **Dynamic bosonic susceptibility at the maximum $T_{\mathbf{c}}$.** Dynamic bosonic susceptibility $\chi(|\mathbf{q}|, \omega = 0)$ at $U = 6$ with $T = 0.1$ ($\beta = 10$) for system sizes $L = 12, 14, 16, 18$. The solid line is a guide to the eye of $\chi(|\mathbf{q}|) \sim 1/|\mathbf{q}|^2$. One sees that the power-law nicely captures the scaling behavior of data.

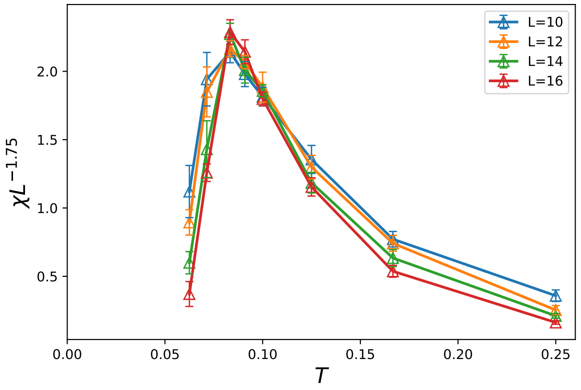

Supplementary Figure 6. **Rescaled bosonic static susceptibility at the re-entry regime.** Rescaled bosonic static susceptibility at $U = 5.9$ versus temperature for system sizes $L = 12, 14, 16, 18$. Quasi-long-range order is expected to exist at the temperature where the rescaled susceptibility increases with system size, otherwise, in the disorder phase.

range order of the s-wave pairing state. This can be seen in Fig. 2b in the main text, where the data collapse of the pairing susceptibility P_{s} using KT phase transition critical characters for different system sizes are presented. One sees that at the temperature scale $T_{\mathrm{c}} \approx 0.05$, a power-law divergence of P_{s} is established. These criteria, together with the results from DOS and ω_n-dependence bosonic dynamic susceptibility, reveals two distinct temperature/energy scales of the fermionic SC properties.

In addition, at $U = 6$, the momentum dependence $\chi(|\mathbf{q}|, \omega = 0)$, falls nicely with the power-law of $|\mathbf{q}|^{-2}$, as shown in Supplementary Figure 5, which is similar to the bare rotor model in Supplementary Equation (25).

B. Scans at the re-entry regime.

In this section, we focus at $U = 5.9$, where the re-entry phenomenon is detected from the boundary

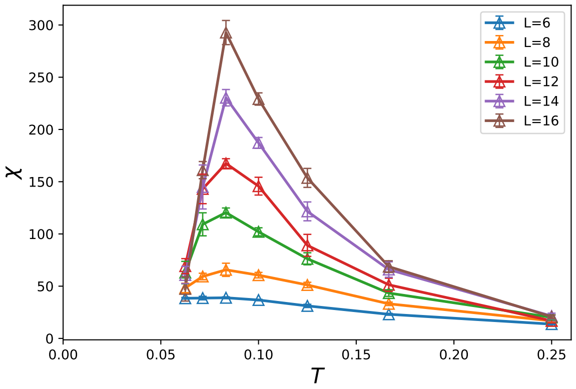

Supplementary Figure 7. **Bosonic static susceptibility at the re-entry regime.** Bosonic static susceptibility $\chi(\mathbf{q}=0,\omega=0)$ versus temperature at $U=5.9$ for system sizes $L=6,8,10,12,14$. Considering finite size effect, the nonmonotonous behavior of $\chi(T)$ depicts a crossover of two phase, whose transition temperature is approxiametly at $T=0.09$.

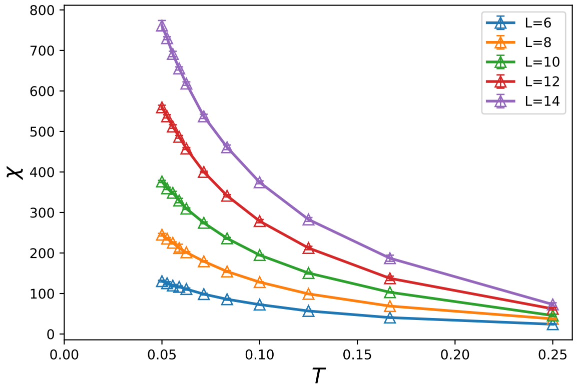

Supplementary Figure 8. **Bosonic static susceptibility at ferromagnetic phase.** Bosonic static susceptibility $\chi(\mathbf{q}=0,\omega=0)$ versus temperature at $U=5.5$ for system sizes $L=6,8,10,12,14$. The nonmonotonous behavior of $\chi(T)$ is not observerd at the lowest temperature $T=0.05$ calculated, indicating the pseudogap crossover temperature is lower than that.

of KT phase transition. We analyze the data in the KT phase at smaller U versus T near the QCP. We first use the scaling of the uniform bosonic susceptibility to determine the U_{KT} as $\chi(U)=L^{2-\eta}f(L\cdot\exp(-\frac{A}{(U-U_{\mathrm{KT}})^{1/2}})$ at fixed T with $\eta=1/4$. It is expected that if one scales $\chi L^{-7/4}$, the curves of different system sizes will cross at the U_{KT} and this is indeed what we saw in Supplementary Figure 6. Here we fix $U=5.9$ and show the uniform susceptibility with different temperature. The KT scaling of the uniform susceptibility manifests, signifying the establish of the quasi-long-range order of the ferromagnetic rotor degrees of freedom. At $T\gtrsim 0.1$ and $T\lesssim 0.08$, the system is obviously located in the disordered phase. At intermediate temperatures at $\beta=11,12$ as calculated, there is clear evidence of forming quasi-long-range order. Therefore, as a function of temperature, the system undergoes two KT phase transitions, i.e., shows the re-entry phenomenon.

Furthermore, the uniform susceptibility also manifests the bending behavior along the temperature axis at $\beta=12$ shown in Supplementary Figure 7.

C. Scans at ferromagnetic phase.

When U is decreased from the QCP to smaller values, the ferromagnetic properties of the bosonic part gradually increase. The pseudogap phase is strongly suppressed, and the crossover temperature drops to the temperature lower than we could explore. Similar to previous method, this can be seen from the bosonic static susceptibility. Supplementary Figure 8 shows that at $U=5.5$, the crossover temperature is lower than $T=0.05$. Thus we think the pseudogap phase disappears quickly at small U in the ferromagnetic regime, as shown in the phase diagram in the main text.

D. Scans in the disordered phase.

The pseudogap phase found in the vicinity of QCP extends to the disordered phase at larger U. Here we present a temperature scan at $U=8$ with $N(\omega)$ at different temperatures. The behavior in Supplementary Figure 9 is similar with that in Fig. 2a in the main text, only that the onset of pseudogap now happens at slightly lower temperature of $T\sim 0.08$. However, the superconducting phase is clearly happening at a much lower temperature compared with that in QCP. For here even with $\beta=24$ the full gap is still not opened, in sharp constrast with the corresponding curve in phase diagram in the main text. Thus we think the SC phase domed at QCP does not extend as much as the pseudogap phase at large U, as shown in the phase diagram in the main text.

E. Scans for the diamagnetic fluctuations.

Many experiments have observed the strong diamagnetic susceptibilities accompanying with pseudogap region. In the lattice model, the diamagnetic fluctuations corresponding to the Meissner effect are identified by measuring the superfluid density ρ_s [15, 16]. This quantity parametrizes the rigidity of the phase of the superconducting order parameter, which natu-

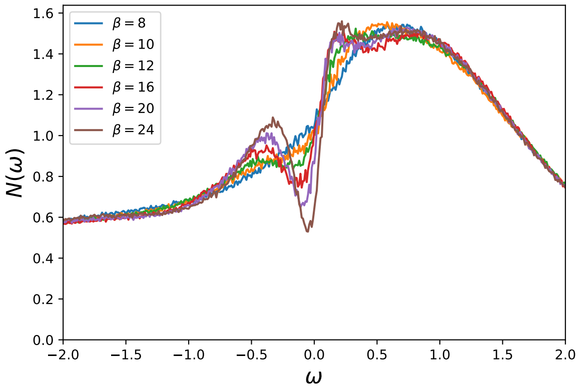

Supplementary Figure 9. **Local DOS in the disordered phase.** Local DOS $N(\omega)$ for various temperature at $U = 8.0$ with $L = 12$. The onset temperature of pseudogap phase is approxiametly at $T = 0.08$. While at the lowest temperature at $T = 0.04$, $N(\omega = 0)$ is still far from 0, indicating the SC phase boundary is far less than $T = 0.04$.

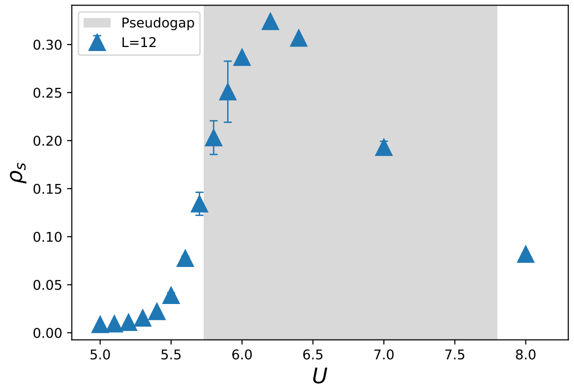

Supplementary Figure 11. **Superfluid density demonstrating diamagnetic fluctuation.** Superfluid density ρ_s versus U at inverse temperature $\beta = 6$ for system size $L = 8, 10, 12$. The grey area corresponds to the pseudogap region for fixing temperature. ρ_s is significantly larger than 0 in the whole pseudogap region, denoting the strong diamagnetic fluctuation.

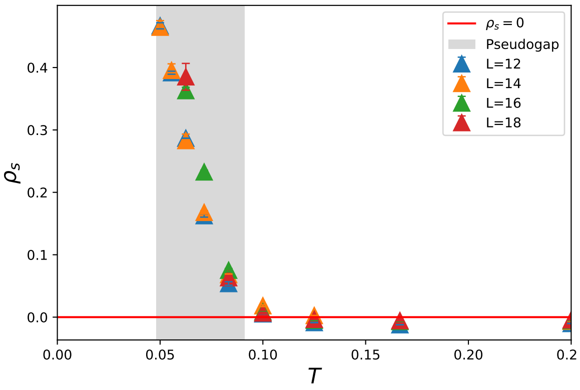

Supplementary Figure 10. **Superfluid density demonstrating diamagnetic fluctuation.** Superfluid density ρ_s versus temperature at $U = 6$ for system size $L = 12, 14, 16, 18$. The grey area corresponds to the pseudogap region for fixing U. The red line denotes $\rho_s = 0$, determining the crossover point for $T_{\rm dia}$, where the finite size effect is negligible. $T_{\rm dia} \approx 0.12$ for $U = 6.0$.

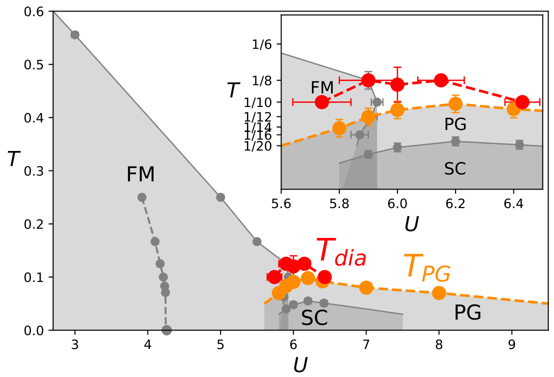

Supplementary Figure 12. Boundary of the diamagnetic fluctuation in the phase diagram. Phase diagram same as which in Fig. 1b in the main text. Other unconcerned regions and boundaries are colored by grey, except the boundary of $T_{\rm PG}$, colored by orange. The boundary of $T_{\rm dia}$ is shown by red. The points on the boundary is determined by the crossover method mentioned above, with fixed U or temperature. The onset temperature of diamagnetism is obviously higher than the onset of pseudogap.

rally leads to measuring the capacity of the superconductor to expel electromagnetic fields. We regard the value of ρ_s goes from negative to positive as changing from paramagnetism to diamagnetism, and the curve consisting of such crossover points is identified as $T_{\rm dia}$ [15, 16]. Specifically, this behavior is observed by fixing the tuning parameter U, and scanning the temperature in Supplementary Figure 3. Here, we put the same data set as that in Supplementary Figure 10, but just reset the range of the y-axis to show the crossover with x-axis more clearly. The putative pseudogap region at $U = 6$ is colored by grey, in which one can see the strong diamagnetic fluctuations, even extrapolating to infinite system size. The pseudogap region is totally under by the $T_{\rm dia}$.

Besides, we fix the inverse temperature $\beta = 16$,

which is above the maximum of T_c, and draw ρ_s versus U in Supplementary Figure 11. The putative pseudogap region is also colored by grey. One can see ρ_s is large enough denoting the existing diamagnetic fluctuations before onset of true SC. Furthermore, we draw a boundary in the phase diagram representing the onset of diamagnetic fluctuations where ρ_s changes sign, marked T_{dia} in Supplementary Figure 12 below. The boundary of T_{dia} covers the whole pseudogap region, and has the similar characters as T_{PG}.

Supplementary Note 4: Theoretical analysis.

A. Modified Eliashberg theory.

We analyzed the QMC data for fermionic and bosonic response using the modified Eliashberg theory (mET), which is a low energy effective dynamical theory for itinerant fermions near a QCP at finite temperatures. Within this theory, one obtains and solves the set of self-consistent equations for fermionic self-energy $\Sigma(k, \omega_n) \approx \Sigma(\omega_m)$ and bosonic propagator $\Pi(q, \Omega_n)$, related to fermionic Green's function $G(k, \omega_n)$ and bosonic susceptibility $\chi(q, \Omega_n)$ as

$$G^{-1}(\mathbf{k}, \omega_n) = i\omega_n - \varepsilon_{\mathbf{k}} + i\Sigma(\mathbf{k}, \omega_n), \quad (26)$$

$$\chi^{-1}(\mathbf{q}, \Omega_n) = \chi_0^{-1}\left(r(T) + q^2 + c^2\Omega_n^2 + \Pi(\mathbf{q}, \Omega_n)\right). \quad (27)$$

The input parameters for mET are

$$k_F, v_F, c, \chi(T) = \frac{\chi_0}{r(T)}, \bar{g}. \quad (28)$$

These are, respectively, the fermionic Fermi vector, Fermi velocity, bosonic velocity, static bosonic susceptibility, and the effective static boson-fermion vertex. At a QCP, $r(T) \to 0$. At the bare level, $\bar{g} = K^2\chi_0$, see Eq. (1) in the main text, but it get substantially renormalized by fermions with energies of order of the bandwidth, and we treat $\bar{g}$ as a fitting parameter. The details of mET approach have been discussed in Refs. [17, 18], and we refer an interested reader to those works for details.

The fermionic self energy consists of a thermal contribution $\Sigma_T(\omega_n)$ and quantum contribution $\Sigma_Q(\omega_n)$,

$$\Sigma(\omega_n) = \Sigma_T(\omega_n) + \Sigma_Q(\omega_n). \quad (29)$$

The thermal contribution is a solution of a self-consistent equation,

$$\Sigma_T(\omega_n) = \frac{\bar{g}T}{\pi}\frac{\mathcal{S}(\mathcal{A}_n),}{|\omega_n + \Sigma_T(\omega_n)|} \quad (30)$$

where (for $n \geq 0$), $\mathcal{A}_n = \frac{v_F\sqrt{r(T)}}{|\omega_n+\Sigma_T(\omega_n)|}$ and $\mathcal{S}(x) = \frac{\cosh^{-1}(1/x)}{\sqrt{1-x^2}}$. The quantum contribution is

$$\Sigma_Q(\omega_n) = \frac{\bar{g}T}{\pi}\sum_{m\neq n}\frac{\mathcal{T}(\mathcal{A}_m, \mathcal{B}_{mn})}{|\omega_m + \Sigma_T(\omega_m)|}, \quad (31)$$

where $\mathcal{B}_{mn} = \frac{(\bar{g}k_F v_F|\omega_n-\omega_m|/\pi)^{1/3}}{|\omega_m+\Sigma_T(\omega_m)|}$ and

$$\mathcal{T}(x, y) = \int_0^\infty \frac{z^2 dz}{\sqrt{z^2+1}(z^3 + zx^2 + y^3)}. \quad (32)$$

The expressions are cumbersome, but allows one to straightforwardly compute Σ_T and Σ_Q numerically. The outcome of the computations is the following. At $T \to 0$, Σ_T vanishes and $\Sigma_Q \propto \omega_m^{2/3}$, leading to the well-known nFL fermionic behavior and $z = 3$ dynamical scaling. At a finite T, there exists a wide range of temperatures, where the variations of Σ_T and Σ_Q with ω_n nearly compensate one another, leading to a fairly flat total self energy $\Sigma(\omega_m)$. Roughly, this happens because Σ_T (Σ_Q) are decreasing (increasing) functions of ω_n.

The bosonic self-energy has the form,

$$\Pi(\mathbf{q}, \Omega_n) = \frac{2\bar{g}Tk_F}{v_F}\times \sum_{m=-n}^{-1}\frac{1}{\sqrt{(\Omega_n + |\Sigma(\omega_{n+m})| + |\Sigma(\omega_m)|)^2 + v_F^2q^2}} \quad (33)$$

For $v_Fq \gg \omega_n, \Sigma$, and at low T, $\Pi \propto \Omega_n/(v_Fq)$ has the form of a canonical Landau damping of the bosons. This gives rise to $z = 3$ scaling. However, at $q = 0$ and at a small but finite Ω_n,

$$\Pi \propto \frac{\Omega_n}{\Sigma_T(\omega_0)}, \quad (34)$$

and the scaling changes to $z = 2$.

Supplementary Equation (33) is justified for small/moderate frequencies. At larger Ω_n, vertex corrections play a role [14, 19, 20]. In the cases when a boson represents a conserved quantity, vertex corrections remove the dependence of $\Pi(q, \Omega)$ on the self-energy, as required by the Ward identity. However, in almost all QMC simulations to date, the boson is not a conserved quantity, and therefore the effect of vertex corrections must be computed in case-by-case basis. The results are that for an Ising spin, ladder vertex corrections are strong, and the dressed

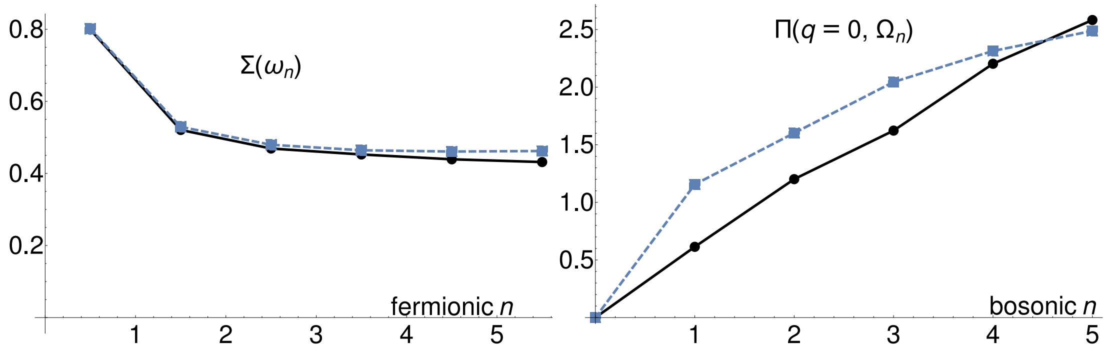

Supplementary Figure 13. **Comparison of QMC data with MET predictions.** (a) The fermionic self energy along the FS diagonal for $U = 6, T = 0.1$. The black circles are QMC data, and the blue squares are the theoretical prediction. (b) The bosonic self energy (for $\mathbf{q} = 0$).

$\Pi(q,\Omega) \propto \Omega_n/\sqrt{\Omega_n^2 + v_F^2 q^2}$, obeys an "effective" Ward identity. Any violation of this identity must arise from additional diagrams, e.g. Aslamasov-Larkin diagrams, which are expected to be weak. For an SU(2) spin, vertex corrections are also strong, but do not cancel out the dependence of Π on Σ. In this situation, Eq. (33) is good only for order of magnitude estimates for $\Omega_n \gtrsim v_F q$. The case of an SO(2) spin, which we have in our simulations, is much better in this regard because ladder vertex corrections actually vanish. In this situation, corrections to Supplementary Equation (33) only come from non-ladder diagrams. These are normally quite small diagrams, so we expect Supplementary Equation (33) to be a fairly decent approximation.

B. Data Analysis.

Since MET is valid in the vicinity of a QCP, we picked data for $U = 6$ to perform the analysis. This is because it is near the critical $U = 5.9$, but the reentrance effect, shown in Fig. 1 in the main text, is weaker there. Nevertheless, the system develops both a PG and magnetic order for low T. This poses several constraints. First, we are limited to $T > 0.1$ to avoid a significant pseudogap. This means that there are very few data points that are valid for a low-energy theory. We picked as a cutoff in frequency $\omega_F = k_F v_F/2$, and as a cutoff in momentum $q_F = v_F^{-1}$, which leaves about 5 Matsubara frequencies within our window at $T = 0.1$. Second, the presence of even a small Zeeman gap distorts the self energy such that the self-energy obeys

$$\Sigma_{FM} = \Sigma(\omega_n) + \frac{\Delta_{FM}^2}{\omega_n} \qquad (35)$$

where $\Sigma(\omega_n)$ appears in Supplementary Equation (29). In terms of the inputs to our theory, Supplementary Equation (28), $k_F, v_F, \chi_0, r(T)$ were obtained from the band structure and from the QMC data for the bosonic propagator. Unfortunately, we were not able to extract c reliably from the data, and so in our calculations we set $c = 0$ for simplicity (we checked that varying c doesn't qualitatively change our results). We then fit the fermionic self energy at the FS to Supplementary Equation (35), using Δ_{FM} and $\bar{g}$ as fitting parameters. We present the data for Σ_{FM} along the BZ diagonal in Supplementary Figure **13**(a), showing excellent agreement with the data. The fit parameters were

$$\Delta_{FM} = 0.34 \pm 0.01, \bar{g} = 6.3 \pm 0.2. \qquad (36)$$

Δ_{FM} is on order of an inverse lattice vector $\pi/L, L = 12$, consistent with the splitting seen in e.g. Fig. 3(b) in the main text. $\bar{g}$ is a bit higher than the bare $\bar{g}_0 = 4.2$ obtained from the model parameters, and represents about a 20% increase in the interaction vertex K, consistent with previous QMC at strong coupling. We expect the coupling in Supplementary Equation (36) to be somewhat over-estimated because we neglected $c > 0$ effects. We checked our fits by comparing the theoretical and QMC bosonic self-energy. We present the comparison in Supplementary Figure **13**(b), which shows a fairly good agreement. The quantitative discrepancies are not surprising, both because of the Zeeman gap and due to the issues discussed above and in the background section. For our purposes, it is enough that the theory correctly predicts the deviation from $z = 3$ scaling, and that the slope of the theoretical and QMC data are comparable, confirming that our estimate of $\bar{g}$ is reasonable.

To confirm that the onset of the pseudogap in our simulations is consistent with theoretical predictions, we computed $T_{\rm PG}$ within the $\gamma-$model (see Ref. [21] and references within). To facilitate comparison with previous works, we supply here the conversion between the coupling $\bar{g}$ in our model, and the effective coupling $\bar{g}_\gamma$ in the $\gamma-$model. It is,

$$\bar{g}_\gamma = \frac{1}{2\pi^2}\frac{\bar{g}^2}{k_{\rm F}v_{\rm F}}\left(\frac{2}{3\sqrt{3}}\right)^3 . \quad (37)$$

Our model corresponds to a $\gamma = 1/3$ model, for which $T_{\rm PG} = 4.4\bar{g}_\gamma$. Using the extracted $\bar{g}$ from Supplementary Equation (36) we find $T_{\rm PG} = 0.08$, which is in good agreement with the measurement of $T_{\rm PG} \sim 0.1$.

[1] W. Jiang, G. Pan, Y. Liu, and Z. Y. Meng, Solving quantum rotor model with different Monte Carlo techniques, Preprint at https://arxiv.org/abs/1912.08229 [cond-mat.str-el].
[2] R. Blankenbecler, D. J. Scalapino, and R. L. Sugar, Monte carlo calculations of coupled boson-fermion systems. i, Phys. Rev. D **24**, 2278 (1981).
[3] X. Y. Xu, Z. H. Liu, G. Pan, Y. Qi, K. Sun, and Z. Y. Meng, Revealing fermionic quantum criticality from new monte carlo techniques, Journal of Physics: Condensed Matter **31**, 463001 (2019).
[4] F. F. Assaad, Depleted kondo lattices: Quantum monte carlo and mean-field calculations, Phys. Rev. B **65**, 115104 (2002).
[5] Y. Wang and A. V. Chubukov, Superconductivity at the onset of spin-density-wave order in a metal, Phys. Rev. Lett. **110**, 127001 (2013).
[6] M. A. Metlitski, D. F. Mross, S. Sachdev, and T. Senthil, Cooper pairing in non-fermi liquids, Phys. Rev. B **91**, 10.1103/PhysRevB.91.115111 (2015).
[7] E. B. Samuel Lederera, Yoni Schattner, Superconductivity and non-fermi liquid behavior near a nematic quantum critical point, Proceedings of the National Academy of Sciences **114**, E8798 (2017).
[8] T. Paiva, R. R. dos Santos, R. T. Scalettar, and P. J. H. Denteneer, Critical temperature for the two-dimensional attractive hubbard model, Phys. Rev. B **69**, 184501 (2004).
[9] N. C. Costa, T. Blommel, W.-T. Chiu, G. Batrouni, and R. T. Scalettar, Phonon dispersion and the competition between pairing and charge order, Phys. Rev. Lett. **120**, 187003 (2018).
[10] C. Chen, T. Yuan, Y. Qi, and Z. Y. Meng, Fermi arcs and pseudogap in a lattice model of a doped orthogonal metal, Phys. Rev. B **103**, 165131 (2021).
[11] D. J. Scalapino, S. R. White, and S. C. Zhang, Superfluid density and the drude weight of the hubbard model, Phys. Rev. Lett. **68**, 2830 (1992).
[12] D. J. Scalapino, S. R. White, and S. Zhang, Insulator, metal, or superconductor: The criteria, Phys. Rev. B **47**, 7995 (1993).
[13] D. R. Nelson and J. M. Kosterlitz, Universal jump in the superfluid density of two-dimensional superfluids, Phys. Rev. Lett. **39**, 1201 (1977).
[14] A. V. Chubukov, J. J. Betouras, and D. V. Efremov, Non-landau damping of magnetic excitations in systems with localized and itinerant electrons, Phys. Rev. Lett. **112**, 037202 (2014).
[15] Y. Schattner, M. H. Gerlach, S. Trebst, and E. Berg, Competing orders in a nearly antiferromagnetic metal, Phys. Rev. Lett. **117**, 097002 (2016).
[16] M. H. Gerlach, Y. Schattner, E. Berg, and S. Trebst, Quantum critical properties of a metallic spin-density-wave transition, Phys. Rev. B **95**, 035124 (2017).
[17] A. Klein, A. V. Chubukov, Y. Schattner, and E. Berg, Normal state properties of quantum critical metals at finite temperature, Phys. Rev. X **10**, 031053 (2020).
[18] X. Y. Xu, A. Klein, K. Sun, A. V. Chubukov, and Z. Y. Meng, Identification of non-fermi liquid fermionic self-energy from quantum monte carlo data, npj Quantum Materials **5**, 65 (2020).
[19] A. V. Chubukov, Ward identities for strongly coupled eliashberg theories, Phys. Rev. B **72**, 085113 (2005).
[20] D. L. Maslov and A. V. Chubukov, Optical response of correlated electron systems, Reports on Progress in Physics **80**, 026503 (2016).
[21] Y.-M. Wu, A. Abanov, Y. Wang, and A. V. Chubukov, Interplay between superconductivity and non-fermi liquid at a quantum critical point in a metal. ii., Phys. Rev. B **102**, 024525 (2020).
